# Modeling Impact-induced Failure of Polysilicon MEMS: A Multi-scale Approach

**DOI:** 10.3390/s90100556

**Published:** 2009-01-19

**Authors:** Stefano Mariani, Aldo Ghisi, Alberto Corigliano, Sarah Zerbini

**Affiliations:** 1 Politecnico di Milano, Dipartimento di Ingegneria Strutturale, Piazza Leonardo da Vinci 32, 20133 – Milano, Italy; 2 MAHRS Unit, STMicroelectronics, Via Tolomeo 1, 20010 – Cornaredo, Italy

**Keywords:** inertial MEMS sensors, impact-induced failure, dynamic fracture, multi-scale FE analysis

## Abstract

Failure of packaged polysilicon micro-electro-mechanical systems (MEMS) subjected to impacts involves phenomena occurring at several length-scales. In this paper we present a multi-scale finite element approach to properly allow for: (i) the propagation of stress waves inside the package; (ii) the dynamics of the whole MEMS; (iii) the spreading of micro-cracking in the failing part(s) of the sensor. Through Monte Carlo simulations, some effects of polysilicon micro-structure on the failure mode are elucidated.

## 1. Introduction

**Figure 1.**
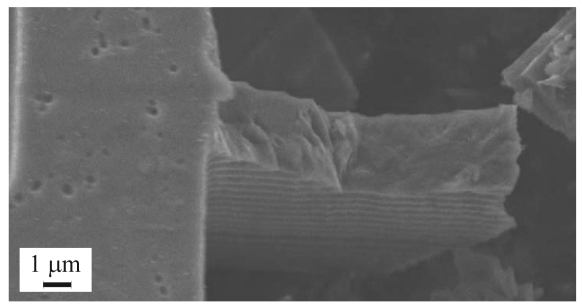
Detail of a shock-induced failure of a suspension spring of a polysilicon MEMS.

Localized failures of polysilicon, inertial MEMS induced by accidental drops and impacts are still a major issue in the assessment of micro-system reliability. Shock-induced failures have been recently investigated, both from experimental and theoretical perspectives, in [1-4], but an accurate description of the failure mechanisms in the polysilicon film (see Figure 1) is far from being attained.

Accuracy of the numerical solutions is typically affected by the presence of at least three length-scales (along with the relevant time-scales) in the failure process. At the package length-scale the propagation of stress waves inside the whole device needs to be accurately tracked; at the sensor length-scale the link between the anchor point motion and the sensor dynamics needs to be established, so as to figure out where the stress state may exceed the polysilicon strength and cause localized ruptures; at the polysilicon length-scale possible failure mechanisms, consisting of cracking at grain boundaries (GBs) and within grains, need to be modeled.

At variance with former semi-analytical proposals (like, e.g. [2, 4]), we recently offered in [5-8] a fully numerical, multi-scale approach to model purely mechanical shock-induced MEMS failure. This approach consists of finite element simulations at each aforementioned length-scale: dissipative phenomena within the anisotropic, heterogeneous polysilicon can therefore be taken in due account. We showed that even unexpected experimental evidences, like e.g. an increase of drop-induced failure occurrence when a uni-axial accelerometer is packaged, can be captured (see [8]).

In this work we furnish a brief description of the multi-scale approach, so as to highlight how accidental drops of micro-systems can be properly modeled; additional details can be found in [5,7,8]. To assess the effect of polysilicon morphology on the possible failure mode of a commercial off-the-shelf inertial sensor, we assumed drop features (like e.g. drop height) to be deterministic; on the other hand, we performed Monte Carlo simulations at the micro-scale (at the polysilicon level) so as to allow for: uncertainties in the orientation of silicon grains at assigned morphology (described through average shape and size of the grains); presence of defective GBs. It is shown that, independently of the micro-structure, failure (if any) is always localized inside a narrow region around the suspension spring-anchor joint and occurs almost instantaneously, i.e. within 0.1 μs at most.

## 2. Multi-scale analysis of polysilicon MEMS failure

**Figure 2.**
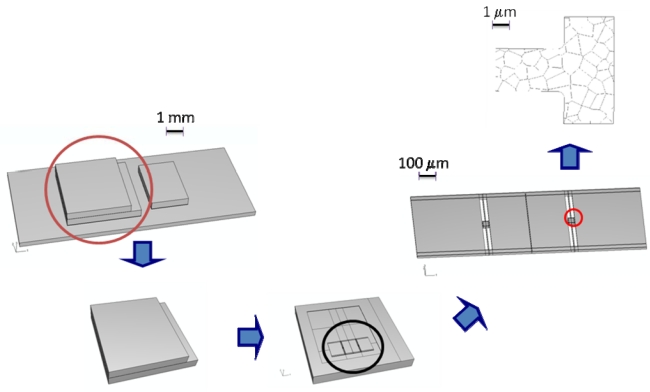
Length scales and domains involved in failure modeling, ranging from macro-scale at the package level down to micro-scale at the polycrystal level.

To accurately model the failure of polysilicon MEMS when subjected to shock loadings, three length-scales are allowed for (see Figure 2):

- Macro-scale. At this scale stress waves propagating inside the whole package need to be tracked; the typical size of the specimens is on the order of a few millimeters at most.
- Meso-scale. At this scale the dynamics of the whole MEMS and the local deformation field in regions close to the anchor points, where the stress field would likely exceed first the polysilicon strength, need to be captured; the size of the specimens is on the order of hundreds of micrometers at most.
- Micro-scale. Because of the brittleness of polysilicon, at this scale the nucleation and subsequent propagation of inter- and trans-granular cracks have to be modeled; the size of the specimens is on the order of micrometers.

While at the macro- and meso-scales all the materials in the device can be considered homogeneous, even if somehow anisotropic, at the micro-scale the morphology of the polycrystal, i.e. the shape and orientation of each silicon grain, must be taken in due account to get a precise picture of the failure mode. Polysilicon is extremely brittle at room temperature [9, 10]: therefore, its failure is highly affected by micro-defects, local orientation of the axes of elastic symmetry of each silicon grain, and statistical distribution of strength and toughness at grain boundaries, see e.g. [11, 12].

As for length-scale interactions, it is worth mentioning that:

- The interaction between macro-scale and meso-scale is negligible in the case here studied. In fact, after the impact this interaction is represented by the tractions transmitted between sensor and package at the anchor points, as resulting from MEMS dynamics; since the mass of the MEMS is three-four orders of magnitude smaller than the package one, the effect of such tractions on the dynamics of the whole device turns out to be negligible.
- The interaction between meso-scale and micro-scale should not be disregarded in principle. In fact, nonlinear processes occurring at the micro-scale directly affect the response of the whole MEMS, up to failure. Because of the brittle behavior of silicon, sensor failure usually occurs almost instantaneously after crack inception: as testified by the forthcoming results, this interaction can be therefore disregarded.

Since length-scale interactions are negligible or can be ignored, the multi-scale approach gets simplified and becomes uncoupled (or hierarchical) [13]: we can thus follow a top-down path. Macro-scale analyses are run to obtain the displacement evolution at the sensor anchors; this evolution is adopted as input at the meso-scale to study sensor shaking. Results of meso-scale analyses are used to identify, on the basis of stress evolution, critical regions which are likely to fail; the evolution of the displacement field at the borders of such regions are adopted as boundary conditions at the micro-scale to obtain forecasts of the failure mode.

**Figure 3.**
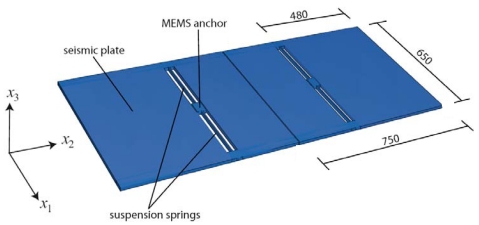
Geometry of the studied uni-axial accelerometers (measures in μm; thickness of the seismic plates is 15 μm).

The capability of the proposed approach is here assessed through a case study. Twin uni-axial accelerometers, whose geometry is depicted in Figure 3, are considered: each seismic plate is anchored to the die through two slender suspension springs. Shock loading is assumed to be caused by an accidental drop of the whole device from a height *h*_drop_ = 1.5 m.

At the meso-scale the interaction between the vibrating seismic plates and the surrounding fluid has been accounted for through proper damping terms in the equations of motion. This interaction was thoroughly investigated in [8], and found to be negligible as for MEMS failure, since failure occurs much before plate dynamics is affected by damping. At this length-scale, seismic plates and suspension springs are considered homogeneous bodies. Their elastic properties are obtained, through an ad-hoc homogenization procedure [5, 8, 14], by exploiting the following features of the polysilicon film they are made of: each silicon grain displays an FCC crystal symmetry; the film texture is perpendicular to the substrate (i.e. it is aligned with axis *x*_3_ in Figure 3); the orientation in the *x*_1_-*x*_2_ plane of the two other axes of elastic symmetry of each grain is randomly distributed. The overall elastic polycrystal response thus turns out to be transversely isotropic, depending on the following five independent parameters: the in-plane (i.e. within plane *x*_1_-*x*_2_) Young's modulus *E* = 152.9 GPa and Poisson's ratio *ν* = 0.2; the out-of-plane Young's modulus *Ē* = 130.1 GPa; the shear modulus *Ḡ* = 79.6 GPa and Poisson's ratio *ν̄* = 0.28, linking in-plane and out-of-plane strain components. Additional details can be found in [5].

As already mentioned, micro-mechanical experimental campaigns [9, 10] showed that polysilicon fails at room temperature because of the nucleation and propagation of inter- as well as trans-granular cracks. Such failure events occur in the studied case within very localized regions, where the stress field is locally amplified by the sensor layout. Depending on polycrystalline details, the failure mode consists of micro-cracking confined along GBs and/or spread within grains. Micro-scale analyses have therefore to allow for the crystal morphology, at least in terms of average grain size and shape, and for the statistics of the orientation in the *x*_1_-*x*_2_ plane of the axes of elastic symmetry of the silicon grains.

**Figure 4.**
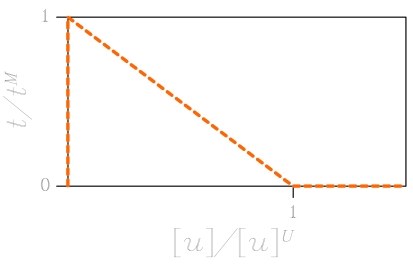
Adopted effective traction *t* – displacement discontinuity [*u*] cohesive envelope.

Micro-cracking evolution in the polysilicon is here simulated through a cohesive approach, whose predictive capabilities in the dynamic regime were shown e.g. in [15-18]. The nucleation of a crack is locally triggered as soon as a norm of the in-plane stress field exceeds the material strength *t^M^* [6, 7]. Afterwards, according to [19], norms of the displacement discontinuity across the crack and of the traction field locally acting on crack faces are respectively defined as:

(1) $$[u]=\sqrt{{\left[u\right]}_{n}^{2}+\beta^{2}{\left[u\right]}_{s}^{2}}$$

(2) $$t=\sqrt{t_{n}^{2}+\frac{1}{\beta^{2}}t_{s}^{2}}$$

where: *β* is a material-dependent model parameter (in the simulations we have adopted *β* = 1); [*u*]*_n_* and [*u*]*_s_* are the opening and sliding displacement discontinuity components, respectively; *t_n_* and *t_s_* are the normal and shear traction components, respectively. The interaction between crack faces is not assumed to abruptly cease; instead, tractions transmitted across faces gradually decrease, following a so-called cohesive envelope, up to the formation of a traction-free crack when the norm [*u*] reaches a critical threshold [*u*]*^J^* (see Figure 4). For computational convenience, this dissipative mechanisms are numerically confined along finite element borders, whereas the bulk material is assumed to always behave elastically; along the whole failure event, energy is thus locally dissipated only for the formation of a traction-free crack.

Because of the layout of the modeled sensor, it results that failure occurs because of the percolation of micro-cracks at the joint section between a suspension spring and the anchor point (see the highlighted detail in the sensor picture, Figure 2): obviously this is not an outcome valid for any MEMS geometry, but instead an effect of the stress concentration due to the presence of the re-entrant corners. Forthcoming results of Monte Carlo simulations are thus confined to that region to get insights into the spreading of micro-cracking and to assess the effect of micro-structural features on the failure mode.

## 3. Effect of polycrystal micro-structure on failure mode: Monte Carlo simulations

To assess the effect of polycrystal features on MEMS failure, results of Monte Carlo simulations are reported henceforth. According to what already shown in [7], two different grain morphologies (termed micro-structures A and B) are considered: they differ in the location of GBs, while grain shape and size have common statistics. At assigned micro-structure, the in-plane orientation of the axes of elastic symmetry of each silicon grain is assumed to be randomly distributed; within a Monte Carlo run at least 100 samples of grain orientations are considered, and outcomes in terms of failure mode and spreading of the cohesive, dissipative processes are treated as random variables too. To assess the possible detrimental effect of defects in the manufacturing process, either perfect or defective GBs are accounted for: assuming fracture toughness Φ = 7.0 J/m^2^ to be homogeneously distributed in space, in the former case trans-granular and inter-granular strengths amount to $t_{\text{G}}^{M}=t_{\text{GB}}^{M}=2.5$ GPa, whereas in the latter case they respectively amount to $t_{\text{G}}^{M}=2.5$ GPa and $t_{\text{GB}}^{M}=2.0$ GPa. As reported in [20], fracture toughness Φ has to depend on the relative orientation of crack plane and crystal lattice; since for silicon the dependence of Φ on the aforementioned orientation leads to a ratio between the maximum toughness variation and the relevant average amounting to a few percent at most, it turns out that allowing for toughness anisotropy does not affect much the failure mode, see [21]. This aspect will be treated in details in a future research.

**Figure 5.**
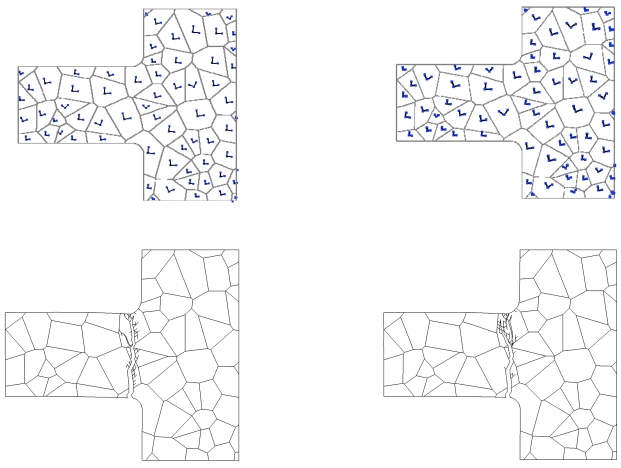
Micro-structure A, perfect GB case ( $t_{\text{G}}^{M}=2.5$ GPa, $t_{\text{GB}}^{M}=2.5$ GPa): effect of crystal morphology on MEMS failure. (top) local orientations of the in-plane axes of elastic symmetry, and (bottom) relevant crack patterns at failure.

**Figure 6.**
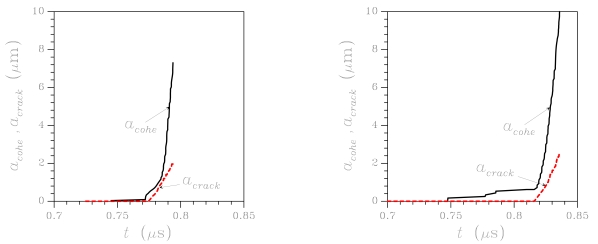
Micro-structure A, perfect GB case ( $t_{\text{G}}^{M}=2.5$ GPa, $t_{\text{GB}}^{M}=2.5$ GPa): effect of crystal morphology (as reported in Figure 5, top row) on the time evolution of crack and cohesive lengths.

To partially understand the links between grain orientation and crack pattern at failure, failure modes relevant to two analyses, wherein micro-structure A with perfect GBs has been adopted, are shown in Figure 5. While the location of cracking initiation is only marginally influenced by grain orientation, it can be clearly noticed that final crack paths look different: in both cases crack branching, which is often observed in dynamic fracture (see, e.g. [22]), shows up; in the second analysis (right column), crack path turns out to be much more tortuous when crossing the uppermost grain. This is revealed also by the time evolution of crack and cohesive zone lengths (the latter being the region where dissipative phenomena is taking place), which are reported in Figure 6 for the same micro-structures shown in Figure 5 (top row): the much wider spreading of micro-cracking in the second analysis is testified by a longer cohesive zone at failure (see the *a*_cohe_ graph).

Figure 6 shows that the inception of dissipative phenomena, i.e. the nucleation of the cohesive zone, is affected by grain orientation; similarly, the propagation of the traction-free crack can be enhanced or delayed by polysilicon morphology. Overall, it can be argued that the whole failure event typically lasts 0.05 – 0.1 μs, depending on grain morphology. Crack tip advancement thus occurs at a speed much lower than that of dilatational waves in polysilicon (on the order of 7–8 km/s): this is mainly due to the branching phenomena occurring along the whole propagation path, which allow to dissipate energy at a low crack tip velocity [22].

**Figure 7.**
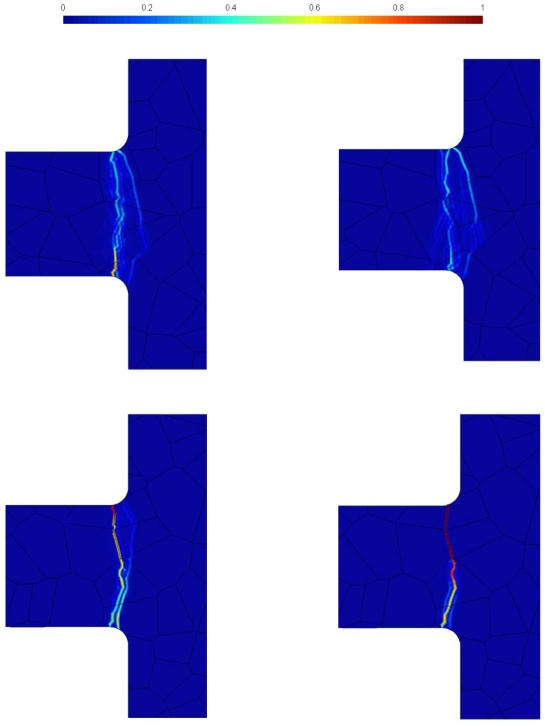
Statistical forecast of the dominant cracks at failure. Top row: micro-structure A; bottom row: micro-structure B. Left column: perfect GBs ( $t_{\text{G}}^{M}=2.5$ GPa, $t_{\text{GB}}^{M}=2.5$ GPa); right column: defective GBs ( $t_{\text{G}}^{M}=2.5$ GPa, $t_{\text{GB}}^{M}=2.0$ GPa).

The previous outcomes suggest that, to thoroughly understand the effects of micro-structure on the failure mode, a statistical analysis is mandatory. Results of Monte Carlo simulations are collected in Figure 7, in terms of forecasts of dominant crack (or cracks) leading to failure. Here the level sets depict the probability for cracking to locally occur: a zero probability means the a crack would never pass through that point, whereas a unitary probability means that a crack will surely pass through. As expected, according to a sort of minimum dissipation principle, cracking is always incepted at a reentrant corner and tends to grow straight, inside a quite narrow region.

As for micro-structure A (top row of Figure 7), a clear tendency to display several alternative crack paths at failure is evidenced, independently of the GB strength: the two crack forecasts thus look spreaded inside the grains closest to the joint section between suspension spring and anchor. If micro-structure B is considered (bottom row of Figure 7), crack is always incepted by the mismatch between grain orientations at the upper spring side: in case of perfect GBs, branching out of the uppermost GB looks possible and a unique crack path at failure cannot be identified; in case of defective GBS, the main crack keeps growing along the network of GBs up to the central portion of the suspension spring, and then it brakes the bottommost grains along an almost straight path, so as to dissipate as less energy as possible, till percolation.

**Figure 8.**
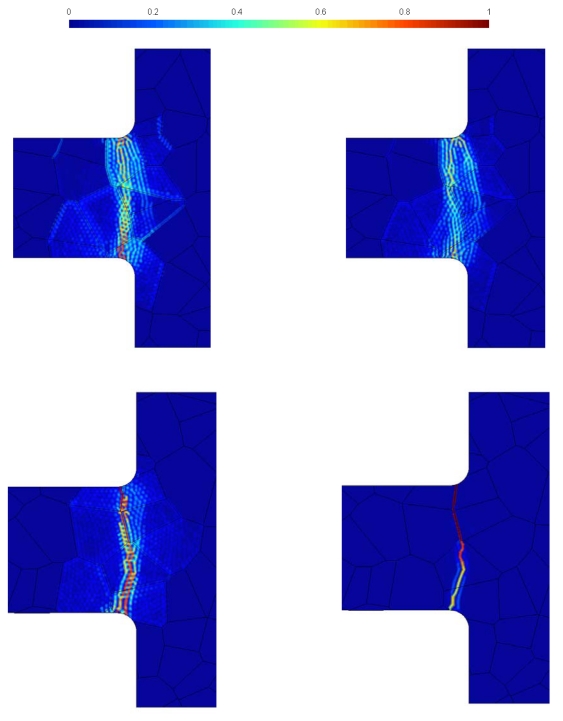
Statistical forecast of the cohesive zone at failure. Top row: micro-structure A; bottom row: micro-structure B. Left column: perfect GBs ( $t_{\text{G}}^{M}=2.5$ GPa, $t_{\text{GB}}^{M}=2.5$ GPa); right column: defective GBs ( $t_{\text{G}}^{M}=2.5$ GPa, $t_{\text{GB}}^{M}=2.0$ GPa).

The same trend is shown in Figure 8 by forecasts of the cohesive zone at failure. While micro-structure A leads to dissipative processes spread around the dominant cracks, micro-structure B shows a tendency to concentrate the cohesive zone very close to the main crack path. This is clearly evident in the defective GB case, featuring forecasts of the cohesive zone and of main crack at failure almost identical, without any scattering in the results.

From these outcomes it turns out that the presence of GBs, specially defective ones, close to the reentrant corners at the joint sections between the suspension springs and the anchor, may be detrimental of the shock-carrying capacity of the sensor. Since micro-structural features (like, e.g., the GB network) can not be controlled during production, an assessment of the effects of shocks and drops on polysilicon MEMS is therefore in need of a statistical analysis, like the one here presented.

## 4. Closing remarks

In this paper a multi-scale, finite element approach has been adopted to assess the effects of polysilicon morphology on the failure mode of an inertial MEMS exposed to shocks. It has been emphasized that, to accurately model such failure process at least three length-scales need to be explored: a macro-scopic one, at the package level; a meso-scopic one, at the sensor level; a micro-scopic one, at the polysilicon level. Thanks to small MEMS inertia and to silicon brittleness, length-scale interactions have been ignored.

Forecasts of cracking pattern at failure have been obtained through Monte Carlo simulations at the micro-scale, at varying polycrystal morphology and in case of perfect/defective grain boundaries. It has been shown that the location of crack inception, and the relevant time elapsed after the impact are affected by micro-structural features. As far as failure is concerned, while the time needed to complete the failure process weakly depends on micro-structural features, the spreading of cracking is highly affected by the network of grain boundaries and by its interaction with the sensor layout, specially close to reentrant corners at the end cross-sections of the suspension springs.
